# The RNA Chaperone Hfq Is Involved in Stress Tolerance and Virulence in Uropathogenic *Proteus mirabilis*

**DOI:** 10.1371/journal.pone.0085626

**Published:** 2014-01-15

**Authors:** Min-Cheng Wang, Hsiung-Fei Chien, Yi-Lin Tsai, Ming-Che Liu, Shwu-Jen Liaw

**Affiliations:** 1 Department and Graduate Institute of Clinical Laboratory Sciences and Medical Biotechnology, College of Medicine, National Taiwan University, Taipei, Taiwan, Republic of China; 2 Department of Surgery, National Taiwan University Hospital, Taipei, Taiwan, Republic of China; 3 Department of Laboratory Medicine, National Taiwan University Hospital, Taipei, Taiwan, Republic of China; Loyola University Medical Center, United States of America

## Abstract

Hfq is a bacterial RNA chaperone involved in the riboregulation of diverse genes via small noncoding RNAs. Here, we show that Hfq is critical for the uropathogenic *Proteus mirabilis* to effectively colonize the bladder and kidneys in a murine urinary tract infection (UTI) model and to establish burned wound infection of the rats. In this regard, we found the *hfq* mutant induced higher IL-8 and MIF levels of uroepithelial cells and displayed reduced intra-macrophage survival. The loss of *hfq* affected bacterial abilities to handle H_2_O_2_ and osmotic pressures and to grow at 50°C. Relative to wild-type, the *hfq* mutant had reduced motility, fewer flagella and less hemolysin expression and was less prone to form biofilm and to adhere to and invade uroepithelial cells. The MR/P fimbrial operon was almost switched to the off phase in the *hfq* mutant. In addition, we found the *hfq* mutant exhibited an altered outer membrane profile and had higher RpoE expression, which indicates the *hfq* mutant may encounter increased envelope stress. With the notion of envelope disturbance in the *hfq* mutant, we found increased membrane permeability and antibiotic susceptibilities in the *hfq* mutant. Finally, we showed that Hfq positively regulated the RpoS level and tolerance to H_2_O_2_ in the stationary phase seemed largely mediated through the Hfq-dependent RpoS expression. Together, our data indicate that Hfq plays a critical role in *P. mirabilis* to establish UTIs by modulating stress responses, surface structures and virulence factors. This study suggests Hfq may serve as a scaffold molecule for development of novel anti-*P. mirabilis* drugs and *P. mirabilis hfq* mutant is a vaccine candidate for preventing UTIs.

## Introduction

The Hfq protein was first identified as a bacterial factor required for the synthesis of bacteriophage Qβ RNA [1]. It belongs to the eukaryotic families of Sm proteins that form homohexameric structures [2]. Hfq is a posttranscriptional regulator that binds small RNAs (sRNAs) and mRNA and facilitates RNA-RNA interaction [2], [3]. Numerous cellular processes, such as stress responses, iron homeostasis and outer membrane protein (OMP) biogenesis are subject to the control of sRNAs and Hfq [1], [4], [5]. For the most part, sRNA-mRNA interactions result in mRNA degradation and/or inhibition of translation. It is now known that Hfq is a small (102 amino acids in *E. coli*), conserved RNA chaperone protein present in many bacterial species [1], [3]. The importance of Hfq became clear when an *E. coli hfq* null mutant was created. This mutant had pleiotropic phenotypes, such as a decreased growth rate, increased sensitivity to cellular stresses, and increased cell length [6].

For most bacteria, *hfq* mutation resulted in diverse phenotypic changes. In striking contrast, deletion of *hfq* in *Staphylococcus aureus* strains did not result in any detectable phenotype [7]. The role of Hfq in the pathogenesis of several bacterial species has been examined [1]. *hfq* mutation in *E. coli* and *Salmonella* Typhimurium results in severe attenuation for virulence [8], [9]. Similarly, decreased virulence was observed for *hfq* mutants of *Pseudomonas aeruginosa*, *Listeria monocytogenes*, *Vibrio cholerae*, and *Yersinia pestis* [10]–[13].

*P. mirabilis* is an important pathogen of the urinary tract, especially in patients with indwelling urinary catheters [14]. Since catheter-associated urinary tract infection (CA-UTI) is a major health concern due to the complications and recurrence, researches directed at understanding the pathogenesis are warranted. The successful colonization of the urinary tract requires that *P. mirabilis* overcome a barrage of innate host defenses, including the shear flow of urine, the antibacterial molecules, the influx of neutrophils, and the generation of reactive oxygen species (ROS) [14], [15]. Common strategies of UTI pathogenesis employed by *P. mirabilis* include fimbria- mediated adhesion and invasion of the uroepithelium, flagella-mediated motility, stress responses, biofilm formation and avoidance of host immune responses [15]. How *P. mirabilis* adapts to ever-changing host milieu is still a mystery. Hfq and sRNA have received considerable attention for their functions in fine-tuning gene expression to facilitate bacterial adaptation. Considering stress tolerance is central to the ability of many bacterial pathogens to successfully colonize hostile host environments and Hfq and sRNAs are key regulators of stress response pathways in other bacteria [1], [4], [5], we were interested in understanding how Hfq might contribute to the virulence of uropathogenic *P. mirabilis*.

Thus far, nothing is known about the role of Hfq in *P. mirabilis*. We employed a mouse model of UTI to show that Hfq is critical for *P. mirabilis* to effectively colonize within the urinary tract. We demonstrated that Hfq affected a number of virulence-related *P. mirabilis* phenotypes, including motility, biofilm formation, and resistance to stresses such as ROS and high osmolarity. In addition, we investigate the correlation of Hfq with RpoS and RpoE. This is the first report about the role of *P. mirabilis* Hfq. This study provides a new insight into the regulation of virulence by Hfq in *P. mirabilis*.

## Materials and Methods

### Ethics statement

All animal experiments were performed in strict accordance to the recommendation in the Guide for the Care and Use of Laboratory Animals of the National Laboratory Animal Center (Taiwan), and the protocol was approved by the Institutional Animal Care and Use Committee of National Taiwan University College of Medicine. All surgery was performed under anesthesia, and all efforts were made to minimize suffering.

### Bacterial strains, plasmids and growth condition

The bacterial strains and plasmids used in this study are listed in Table 1. Bacteria were routinely cultured at 37°C in Luria-Bertani (LB) medium. The LSW^−^ agar, a medium that can inhibit swarming motility of *P. mirabilis* was used for selecting mutant clones and colony counting [16].

**Table 1:** Bacterial strains and plasmids used in this study.

Strain or plasmid	Genotype or relevant phenotype	Source or reference
*Proteus mirabilis*		
wt	Wild-type; Tc^r^	Clinical isolate
hfq	wt derivative; *hfq*-knockout mutant; Km^r^	This study
hfqc	hfq containing pACYC184-*hfq*; Hfq -complemented strain; Cm^r^	This study
hfqca	hfq containing pGEM®-T Easy-*hfq*; Hfq -complemented strain; Amp^r^	This study
rpoS	wt derivative; *rpoS*-knockout mutant; Km^r^	This study
rpoSc	rpoS containing pACYC184-*rpoS*; RpoS -complemented strain; Cm^r^	This study
*E. coli*		
DH5α	*fhuA2 lac(del)U169 phoA glnV44 Φ80' lacZ(del)M15 gyrA96 recA1 relA1 endA1 thi-1 hsdR17*	Invitrogen
S17-1 λ *pir*	λ *pir* lysogen of S17-1 [*thi pro hsdR^−^ hsdM* ^+^ *recA* RP4 2-Tc::Mu-Km::Tn*7* (Tp^r^ Sm^r^)]; permissive host able to transfer suicide plasmids requiring the Pir protein by conjugation to recipient cells	[16]
Plasmids		
pGEM®-T Easy	High-copy TA cloning vector; Amp^r^	Promega
pGEM®-4Z	High-copy cloning vector; Amp^r^	Promega
pUT/mini-Tn*5*-Km	Suicide plasmid requiring the Pir protein for replication and containing a mini-Tn*5* cassette containing Km^r^ gene	[16]
pACYC184	Low-copy cloning vector, P15A replicon; Cm^r^ Tet^r^	[16]
pGEM®-T Easy-*hfq*	pGEM®-T Easy containing intact *hfq* sequence including its ribosome binding site (rbs); Amp^r^	This study
pACYC184-*hfq*	pACYC184 containing intact *hfq* sequence including its ribosome binding site (rbs); Cm^r^	This study
pACYC184-*rpoE- xylE*	*rpoE* reporter plasmid, pACYC184 containing intact *rpoE* promoter sequence before *xylE*; Cm^r^	[16]

### Gene-knockout by homologous recombination

Sequences flanking the *hfq* were amplified by PCR using primer pairs hfqku-F/Xbahfqku-R and Xbahfqkd-F/hfqkd-R (Table 2), respectively and cloned into pGEM®-T Easy (Promega, USA) to generate pGhfq-up and pGhfq-dn. The pGhfq-up was digested with *Sal*I/*Xba*I and the *hfq* upstream sequence-containing fragment was ligated to the *Sal*I/*Xba*I-digested pGhfq-dn to produce the pGhfq-updn plasmid which contains both upstream and downstream sequences of *hfq*. A Km^r^-cassette was inserted to the *Xba*I-digested pGhfq-updn plasmid to generate the pGhfq-updn-Km, a plasmid containing the Km^r^-cassette-disrupted combined upstream and downstream sequence of *hfq*. The DNA fragment containing the Km^r^-cassette-disrupted combined upstream and downstream sequence of *hfq* was cleaved by *Sal*I/*Sph*I from pGhfq-updn-Km and ligated into *Sal*I/*Sph*I-cleaved pUT/mini-Tn*5*-Km [16] to generate pUThfq-Km. Gene inactivation mutagenesis by homologous recombination, and confirmation of the *hfq* mutant with double-crossover events were performed as described previously [16]. *rpoS* mutant was obtained in a similar way.

**Table 2:** Primers used in this study.

Primers	Sequence (5′ to 3′)	Description
hfqku-F	CCGCATTACCTTATTCTG	For *hfq* knockout. Paired with “Xbahfqku-R”.
Xbahfqku-R	TCTAGATACTAGAACCTAATGGTTCG	
Xbahfqkd-F	TCTAGAGGTAGGGAGACTTTACCTATG	For *hfq* knockout. Paired with “hfqkd-R”.
hfqkd-R	GGGTGAAGTCCTCAAGAAG	
rpoSku-F	CAACGACTTCGACACCAAC	For *rpoS* knockout. Paired with “XbarpoSku-R”.
XbarpoSku-R	TCTAGACAGCTGCTCCTACCCTTG	
XbarpoSkd-F	TCTAGAGCTTTAGGTGCTCAATGCG	For *rpoS* knockout. Paired with “rpoSkd-R”.
rpoSkd-R	GCCATTGTTGAAACACCCC	
hfqc-R	CATAGGTAAAGTCTCCCTACC	For *hfq* complementation. Paired with “hfqku-F”.
IL8rt-F	CACACTGCGCCAACACA	For IL-8 real-time RT-PCR. Paired with “IL8rt-R”
IL8rt-R	TCAGCCCTCTTCAAAAACT	
MIFrt-F	AACCGCTCCTACAGCAAG	For MIF real-time RT-PCR. Paired with “MIFrt-R”
MIFrt-R	GTTGTTCCAGCCCACATT	
GAPDHrt-F	CTTTGGTATCGTGGAAGG	Internal control for cytokine real-time RT-PCR. Paired with “GAPDHrt-R”
GAPDHrt-R	GATGATGTTCTGGAGAGC	
rpoSrt-F	GCCTTATTCGTGCTGTTG	For *rpoS* real-time RT-PCR. Paired with “rpoSrt-R”
rpoSrt-R	GACGAATAGTGCGGGTTT	
gyrBrt-F	GACCCGTACGCTAAACAAC	Internal control for *rpoS* real-time RT-PCR. Paired with “gyrBrt-R”
gyrBrt-R	AGAAATAACCGCAATCAGG	
mrpP1	GCATCAATAAAGGGTTGTGTTTT	For the IE assay of the MR/P fimbriae. Paired with mrpP2.
mrpP2	GTAATTGAGCAAGGAGCATCAAT	

### Construction of the Hfq-complemented strain

Full length *hfq* was amplified by PCR using primer pair hfqku-F and hfqc-R (Table 2) and cloned into pGEM®-T Easy (Promega, USA) to generate pGhfq. The DNA fragment containing full length *hfq* was excised from pGhfq with *Sal*I and *Sph*I. The DNA fragment was ligated into a *Sal*I/*Sph*I-digested low-copy plasmid, pACYC184, to generate the *hfq* complementation plasmid, pACYC184-*hfq*. The pACYC184-*hfq* was then transformed into the *hfq*-knockout mutant to generate the Hfq-complemented strain (hfqc). The RpoS-complemented strain (rpoSc) was constructed in a similar way. The pGEM®-T Easy-*hfq* transformed *hfq* mutant was used as the Hfq-complemented strain (hfqca) to prevent mutual exclusion of plasmids only in the *rpoE* reporter assay.

### Mouse infections

The C57BL/6 mouse model of UTI was used as described previously [17], with some modifications. Briefly, 6-week-old female mice were injected transurethrally with a 50-µl overnight culture suspension of *P. mirabilis* strains at a dose of 10^7^ CFU per mouse. On day 3 and day 6 after injection, mice were sacrificed, and bladder and kidney samples were collected, weighed, suspended in 0.5 ml of PBS and then homogenized to determine the viable cell count by plating on LSW^−^ agar plates.

### Rat burned wound infections

Adult male Wistar rats (500–650 g) were anesthetized by intraperitoneal injection of Ketamine plus Rompun. The dorsum of the rat was shaved with hair clippers. For each rat, four burned wounds were created by pressing 1×1 cm^2^ copper plates preheated to 120°C on its back for 12 seconds. The wound area was protected using a polyethylene foam sheet with a 1×1 cm^2^ hole in the middle to create a well for infection. 20 µl of late log bacterial suspension (10^8^ cells/ml) was applied evenly on each wound well and each well was covered with Tegaderm™ transparent dressing (3M™, USA) to prevent contamination by other microbes and to maintain humidity. After overnight infection, the wound was washed with PBS and superficial burned skin was removed, weighed, and homogenized in PBS. Aliquots of homogenates were spread on LSW^−^, and CFU was determined after overnight incubation at 37°C.

### Cytokine array and real-time RT-PCR

Determination of the relative levels of selected human cytokines and chemokines was performed using the Human Cytokine Array Panel A (R&D Systems, USA) according to the manufacturer's instructions. In brief, the human uroepithelial NTUB1 cells [18] were grown in the 12-well plate and incubated with overnight bacterial cultures in RPMI 1640 at a multiplicity of infection (MOI) of 10 (6×10^6^ CFU/well) for 3 h at 37°C. The culture supernatant and NTUB1 cells were subjected to analyses of the cytokine array and real-time RT-PCR, respectively. A mixture of NTUB1 cell culture supernatant and the detection antibody was added to the membrane spotted with capture antibodies. After incubation, the membrane was washed and the chemiluminescence produced by the sample/antibody hybrid on the membrane was detected after adding streptavidin-labeled horseradish peroxidase. For real-time RT-PCR of cytokines (IL-8 and MIF), NTUB1 cells were washed, total RNA was extracted and real-time RT-PCR was performed as described [16] to monitor the expression of IL-8 and MIF mRNA normalized against GAPDH mRNA. To study the effect of the *hfq* mutation on the level of *rpoS* mRNA, overnight cultures of the wild-type, *hfq* mutant and Hfq-complemented strains were washed and total RNA was isolated for real-time RT-PCR.

### Intra-macrophage survival assay

The assay was performed as described before [19], with some modifications. Briefly, mouse macrophage cells (RAW264.7) were cultured to 2×10^6^ cells/well in 12-well plates in RPMI 1640 medium with 10% FBS. The overnight culture of *P. mirabilis* was applied to each well at an MOI of 10. Bacteria were brought in contact with macrophages by centrifugation and incubated for 30 min at 37°C. After infection, the cells were washed with PBS and incubated for 1 h in RPMI 1640 with streptomycin (250 µg/ml) to kill extracellular bacteria. Immediately, some wells were lysed by 1% Triton X-100 to determine the CFU of intracellular bacterial cells at t_0_; others were incubated in medium containing 250 µg/ml streptomycin for additional 2 and 4 h to obtain the respective CFU. The CFU obtained from the lysate of the wild-type-infected macrophages at t_0_ was set as 100%, and other data were relative to this value.

### Stress tolerance assays

For the survival test, overnight cultures were diluted 1∶100 in LB broth, grown to OD_600_ of 0.6, and adjusted to 10^8^ cells/ml in LB broth. Cells were exposed for 20 min to 50°C or 20 mM H_2_O_2_ at 37°C. The numbers of bacteria surviving the stress were measured by colony counting on LSW^−^ agar plates and percent relative cell survival was obtained relative to the untreated control. For high osmolarity growth tests, bacterial cells were prepared the same as in the survival test. Urea and NaCl were added to the bacterial cell suspension at the concentration of 400 mM and 5%, respectively. Bacterial growth was monitored by measuring the OD_600_. To investigate if the RpoS-expressing plasmid can compensate *hfq* mutant for the defect in H_2_O_2_ tolerance, overnight bacterial cultures were used instead in the H_2_O_2_ survival test.

### Swarming and swimming assays

The swarming assay was performed as described previously on 1.5% (w/v) LB agar plates [16]. The swimming migration was determined in a similar way except for incubation for 18 h on 0.3% LB agar plate.

### Assays of the flagellin level and haemolysin activities

Flagellin levels and hemolysin activities were determined as described [16].

### Transmission electron microscopy (TEM)

TEM was performed as described by Caiola *et al.* [20] with some modifications. Overnight bacterial cultures were diluted 1∶100 in LB broth and incubated at 37°C for 4 h. Then, a total of 500 µl bacterial culture was washed and resuspended in 100 µl of PBS, from which 10 µl of the bacterial suspension was applied onto a carbon-coated grid (CF300-Cu, Electron Microscopy Sciences). After 20 min, excess solution was removed by a filter paper. Let the grid dry for additional 10 min. Finally, 1% phosphotungstic acid (PTA, 10 µl) was applied to the grid, staining for 15 sec and excess PTA was removed. On the next day, TEM pictures were obtained with a Hitachi H-7100 electron microscope.

### Biofilm formation assay

Biofilm formation was assayed by measuring the ability of cells to adhere to the wells of 96-well microtitre dishes made of polyvinylchloride (Becton Dickinson, USA) as described [18] with some modifications. Overnight LB cultures were diluted with LB broth and 100 µl was transferred to the microtitre well. The microtitre dishes were sealed and incubated at 37°C for 16 h. After incubation, the wells were rinsed and air dried at room temperature for 15 min. Finally, the crystal violet-stained biofilms were then extracted with 95% ethanol and the absorbance was determined.

### Cell adhesion and invasion assays

Overnight bacterial culture was diluted 100 fold and grown for 3 h. NTUB1 cells prepared [18] were then infected at 37°C for 1.5 h with 1 ml of the bacterial suspension containing 5×10^7^ bacteria. For the adhesion assay, infected monolayers were washed with PBS to remove non-adherent bacteria and lysed with 1% TritonX-100. Cell lysates were serially diluted and plated on LSW^−^ agar plates to determine the total CFU associated with NTUB1 cells (CFUt). For the invasion assay [18], infected NTUB1 cells were washed and further incubated at 37°C for 1.5 h in 1 ml of RPMI 1640 medium containing streptomycin (250 µg/ml) to kill extracellular bacteria. NTUB1 cells were then washed and lysed. Cell lysates were diluted serially and plated on LSW^−^ agar plates to quantify viable invading bacteria (CFUi). Subtraction CFUi from CFUt, we had CFUa (CFU of adherent bacteria). The adhesion ability was expressed as percentage of adherent bacteria versus total inoculum and the invasion ability was expressed as percentage of viable bacteria that survived the streptomycin treatment versus total inoculum.

### The invertible element (IE) assay

The expression of MR/P fimbriae, encoded by *mrp* operon, depends on the promoter orientation [21]. The IE “on” means the promoter direction is for *mrp* operon expression. Genomic DNA of overnight cultures of *P. mirabilis* strains was prepared for amplification of the IE element using mrpP1 and mrpP2 primers (Table 2). The PCR product was digested with *Afl*II and resolved on a 2% agarose gel.

### Analysis of outer membrane proteins

Overnight bacterial cells were broken by a sonicator and membranes were prepared as described previously [22]. The inner membrane was solubilized by adding Sarkosyl NL-97 and the outer membrane fraction was pelleted by centrifugation at 100,000 g for 30 min and assayed by SDS-PAGE.

### Reporter assay

The *rpoE-xylE* reporter plasmid [16]-transformed wild-type, *hfq* mutant and Hfq-complemented strains were grown overnight in LB broth with 20 µg/ml chloramphenicol. The XylE activity was measured as described [16].

### MIC assay

MICs of antibiotics for wild-type *P. mirabilis*, and *hfq* mutant were determined by the broth microdilution method according to the guidelines proposed by the Clinical and Laboratory Standards Institute [23].

## Results

### Deletion of *hfq* from *P. mirabilis*

**Figure 1:**
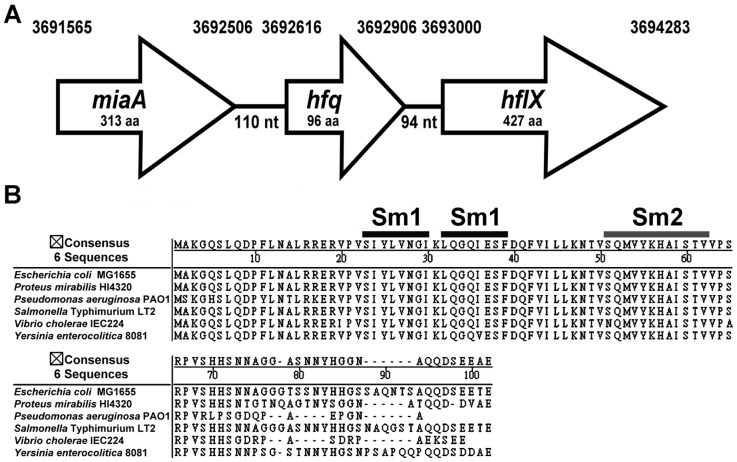
*P. mirabilis hfq* locus and Hfq protein. (**A**) Genomic organization of the *hfq* locus in *P. mirabilis*. The nucleotide numbers of the locus in the genome are listed on the top. (**B**) Alignment of Hfq proteins *of E. coli*, *P. mirabilis*, *P. aeruginosa*, *Salmonella* Typhimurium, *V. cholerae* and *Y. enterocolitica* using the NASTAR- MegAlign program. The highly conserved Sm1 and Sm2 motifs are indicated.

The *hfq* gene was identified at bps 3692616 to 3692906 in the genome of *P. mirabilis* strain HI4320. As in *E. coli*, it is located in the *miaA-hfq-hflX* cluster as indicated in Figure 1A. The Hfq protein consists of 96 amino acids and shares 86% sequence identity with its homologue in *E. coli* (*Proteus* Hfq is identical to *E. coli* Hfq for the first 74 amino acids, with only the C-terminal tail varying), and just like other bacterial Hfq proteins, it has the conserved Sm1 and Sm2 motifs (Figure 1B). In order to examine the role of Hfq in *P. mirabilis*, we generated an isogenic mutant lacking the entire Hfq coding sequence and the Hfq-complemented strain. We confirmed absence of the polar effect on *hflX*, the gene immediately downstream of *hfq*, by real-time RT-PCR (data not shown). The loss of *hfq* in some bacterial species has been shown to have growth defects [12], [13], [24]. We then monitored the growth of the wild-type, *hfq* mutant and the Hfq-complemented strain in the LB broth. The loss of Hfq resulted in a slight growth defect during the first 7-h growth and overnight cultures of wild-type and *hfq* mutant routinely reached the same density (data not shown). On LB agar plates, the colonies formed by *hfq* mutant are more transparent than the wild-type (data not shown).

### *P. mirabilis hfq* mutant was attenuated for colonization of the urinary tract

**Figure 2:**
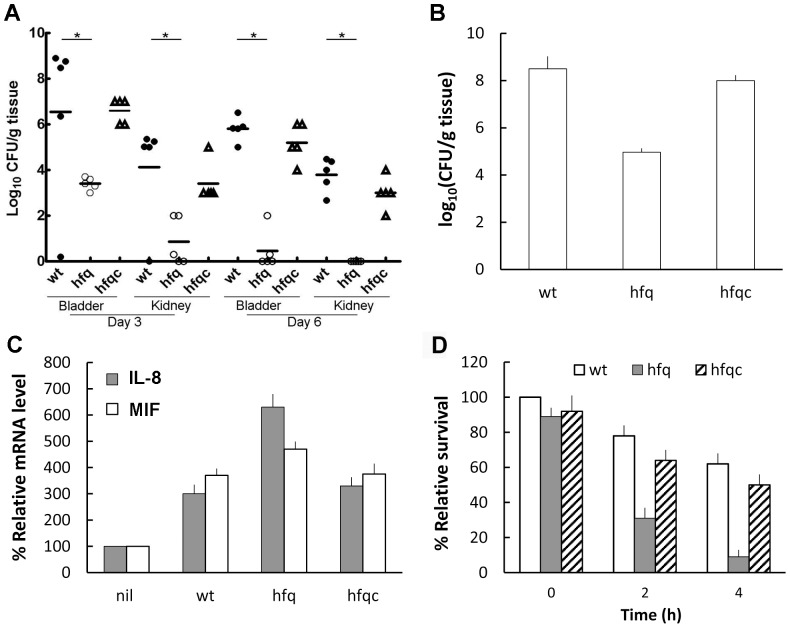
*P. mirabilis hfq* was required for colonization of the urinary tract, infection of the burned wound, induction of cytokine expression and survival in macrophage. (**A**) Colonization of wild-type, *hfq* mutant or the Hfq-complemented strain in the mouse bladder and kidneys. Bacterial loads were determined in the bladder and kidneys on day 3 and day 6 after transurethral inoculation with suspension containing equal amount of bacteria (10^7^ CFU). Horizontal bars indicate median values for each group. Filled and open circles and triangles represent wild-type, *hfq* mutant and Hfq-complemented strain retrieved from five C57BL/6 mice, respectively. *, Significant difference by Student's t-test analysis (*p<0.01). (**B**) Bacterial loads in the burned wounds of rats infected with *P. mirabilis* wild-type, *hfq* mutant or the Hfq-complemented strain. Bacterial loads were determined from the wounds of three Wistar rats after inoculation with suspension containing equal amount of bacteria (2×10^6^ CFU/wound). The data represent the average of three rats with standard deviation. (**C**) *hfq* mutation induced expression of IL-8 and MIF. IL-8 and MIF mRNA levels of NTUB1 cells after challenging with the wild-type, *hfq* mutant or the Hfq-complemented strain were measured by real-time RT-PCR. The value of NTUB1 cells without challenging (nil) was set at 100%. (**D**) Survival of the wild-type, *hfq* mutant or the Hfq-complemented strain in macrophage. RAW264.7 macrophage cells were infected with bacteria for 30 min at an MOI of 10 and intracellular survival of the bacteria was determined by the streptomycin protection assay at 0, 2 and 4 h. The value obtained with the wild-type cells at 0 h after streptomycin treatment was set at 100%. The data represent the average of three independent experiments with standard deviation in **C** and **D**. wt, wild-type; hfq, *hfq* mutant; hfqc, Hfq-complemented strain.

To assess the role of Hfq in UTI caused by *P. mirabilis*, the C57BL/6 mouse model of ascending UTI was used [17]. As shown in Figure 2A, the *hfq* mutant had impaired abilities to colonize within the bladder and kidneys relative to the wild-type. Significant difference in the bacterial load of both the bladder and kidneys was observed between the wild-type and *hfq* mutant on day 3 and day 6 post-inoculation, noting that *hfq* mutant almost can't colonize the kidney on day 6. This result indicates Hfq is required for colonization and survival of *P. mirabilis* in the urinary tract. In addition, we used a rat burned-skin model to demonstrate that the ability to infect the burned-skin was also significantly impaired in the *hfq* mutant. We found the bacterial load recovered from the wild-type-infected skin was significantly higher than that of the mutant (Figure 2B) and the skin area infected by wild-type was severely damaged, compared to the more intact appearance of the area by *hfq* mutant (data not shown). The Hfq-complemented strain displayed behavior comparable to the wild-type in both the mouse UT colonization and the rat skin infection. In order to know how the loss of Hfq could lead to such a severe reduction in the bacterial colonization of the urinary tract and the attenuated burned-skin infection, we then examined key virulence factors of *P. mirabilis* in the following experiments.

### Hfq affected cytokine production of the uroepithelial cells

IL-8 and MIF are key cytokines contributing to bacterial elimination by the host cells and are expressed in the epithelial lining of the skin and the urinary tract [25], [26]. Knowing Hfq plays a role in *P. mirabilis* colonization of bladder and kidney (Figure 2A, 2B), we examined the production of IL-8 and MIF by NTUB1 cells challenged with wild-type or *hfq* mutant. Both IL-8 and MIF were increased significantly in the *hfq* mutant compared to the wild-type in the cytokine array assay (data not shown). Real time RT-PCR (Figure 2C) confirmed that expression of IL-8 and MIF mRNA was increased in the *hfq* mutant relative to the wild-type and the Hfq-complemented strain. This result suggests loss of Hfq may trigger NTUB1 cells to produce higher levels of IL-8 and MIF and then attract the immune cells to confine the bacterial loads in vivo.

### The loss of *hfq* increased killing of *P. mirabilis* by macrophage cells

In order to know if Hfq is involved in the innate ability of macrophages to eliminate *P. mirabilis*, we challenged macrophages with bacteria, killed external bacteria with streptomycin and assessed the survival of internalized *P. mirabilis* after streptomycin treatment by lysing macrophage cells. In general, *P. mirabilis* is assumed not to replicate inside macrophages and thus, in this niche, survival within a short time is an important fitness index [27]. We found there is no significant difference between the intra-macrophage survival of wild-type and *hfq* mutant cells at t_0_. However, the relative survival of *hfq* mutant was reduced to 30 and 10% at 2 and 4 h after streptomycin treatment, respectively, compared to 80 and 60% in the wild-type (Figure 2D). The Hfq-complemented strain exhibited the survival pattern similar to the wild-type.

### Hfq contributed to the resistance to H_2_O_2_, high temperature and high osmotic pressure in *P. mirabilis*

**Figure 3:**
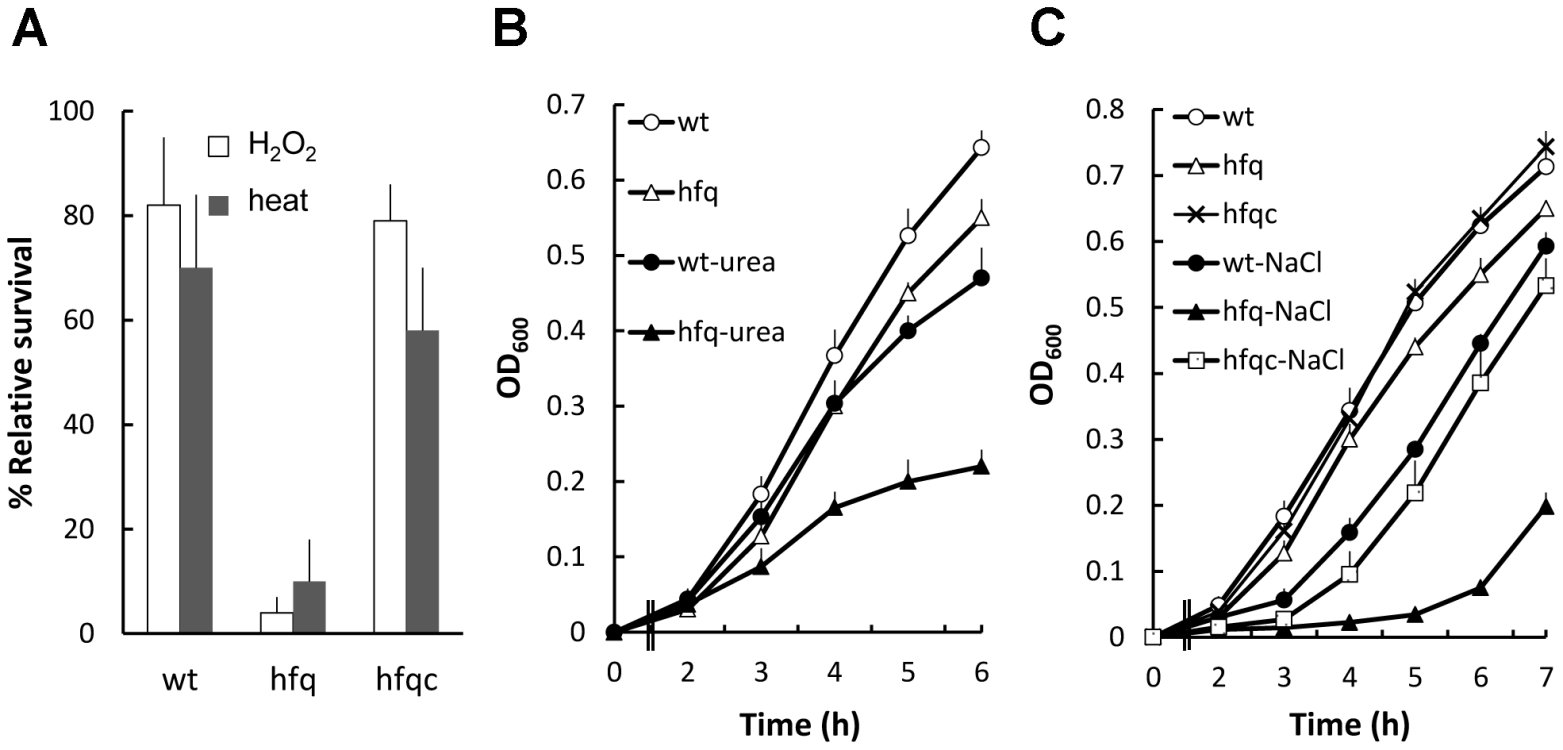
The effect of *hfq* on stress tolerance of *P. mirabilis*. (**A**) Tolerance to H_2_O_2_ and 50°C. Exponentially growing wild-type, *hfq* mutant and Hfq-complemented strain (10^8^ cells/ml in LB broth) were exposed to 50°C or H_2_O_2_ (20 mM, at 37°C) for 20 min. The numbers of bacteria surviving the stress were measured and relative cell survival was obtained relative to the untreated control. (**B**) and (**C**) Tolerance to urea and 5% NaCl. Bacterial cultures of the exponential phase were adjusted to 10^8^ cells/ml in LB broth with or without 400 mM urea or 5% NaCl. Bacterial growth was monitored by measuring the OD_600_. All the data represent the average of three independent experiments with standard deviation. wt, wild-type; hfq, *hfq* mutant; hfqc, Hfq-complemented strain.

To determine if Hfq is required for *P. mirabilis* to cope with stresses it may encounter within the urinary tract during infection, such as oxidative stresses and high osmolarity, the growth of wild-type and *hfq* mutant was assessed in various stressful conditions. The loss of *hfq* caused significantly decreased survival after exposure to 20 mM H_2_O_2_ or 50°C for 20 min (Figure 3A). Furthermore, growth of *hfq* mutant was more significantly impaired relative to the wild type in the presence of 400 mM urea (a major component in urine in a physiologically relevant concentration) or 5% NaCl (Figure 3B, 3C). The reduced tolerance to H_2_O_2_, 50°C or 5% NaCl of *hfq* mutant was restored to near the wild-type level by introduction of the Hfq-expressing plasmid (Figure 3A, 3C). This implies Hfq can provide *P. mirabilis* with protection from injuries of high osmolarity and ROS in the urinary tract.

### The effect of Hfq on motility of *P. mirabilis*

**Figure 4:**
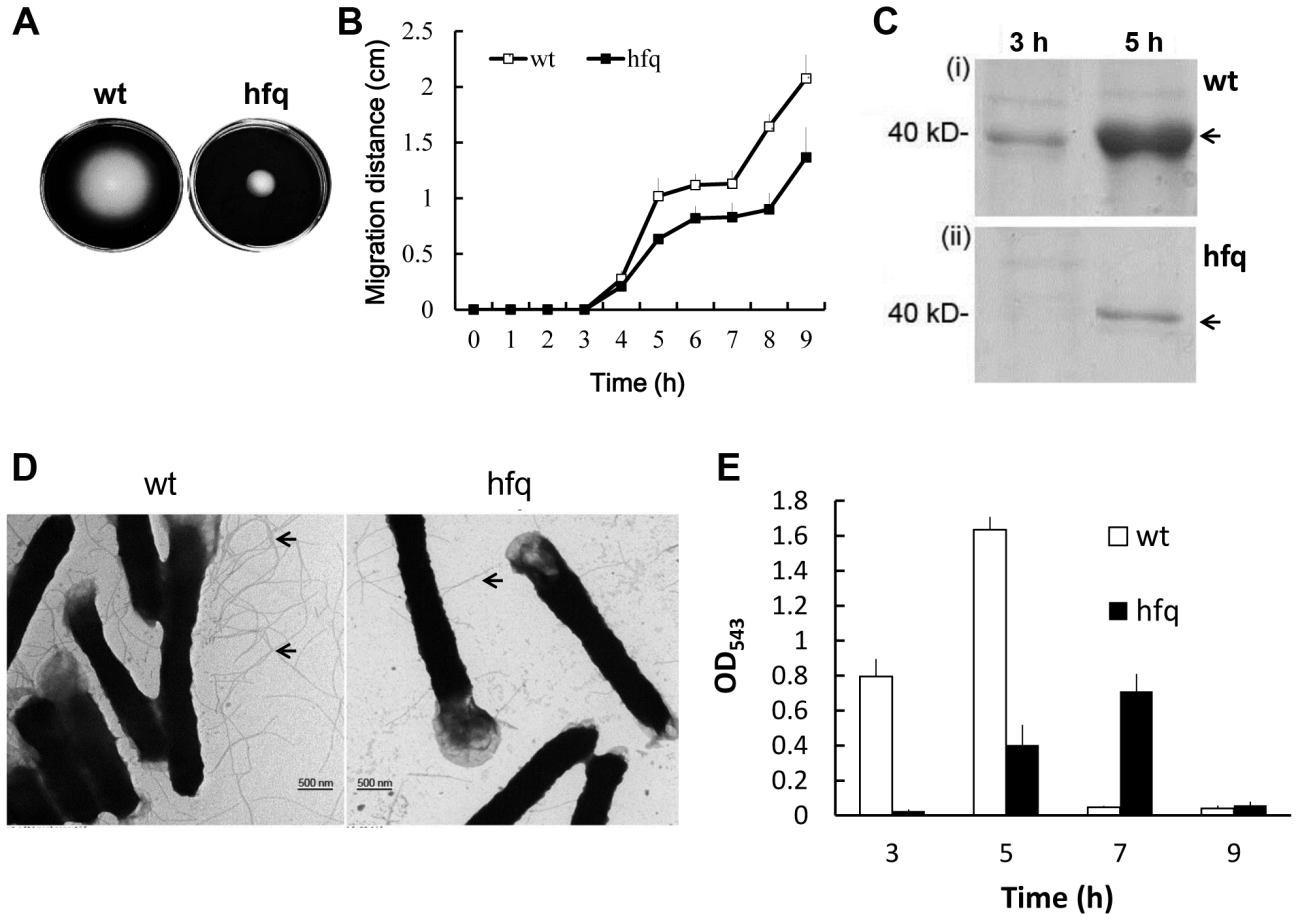
*P. mirabilis hfq* was required for motility and production of flagellin, flagella and hemolysin. (**A**) Swimming motility of the wild-type and hfq mutant. Aliquots (5 µl) of overnight culture were inoculated centrally into the LB swimming plates. The plates were incubated at 37°C. The representative picture was taken at 16 h after inoculation. (**B**) Swarming motility of the wild-type and hfq mutant. Aliquots (5 µl) of overnight culture were inoculated centrally onto the LB swarming plates. The plates were incubated at 37°C and the migration distance was measured hourly after inoculation. (**C**) The SDS-PAGE profile of flagellin of wild-type and *hfq* mutant at 3 h and 5 h after inoculation on swarming plates. The band of flagellin is indicated by an arrow. (**D**) TEM pictures of wild-type and *hfq* mutant cells. Bacterial cultures were applied onto a grid, cells were stained with 1% PTA and pictures were taken. Flagella are indicated by arrows. (**E**) Hemolysin activities of wild-type and *hfq* mutant during the swarming migration process. The representative result from three independent experiments is shown in **A**, **C** and **D**. In **B** and **E**, the data represent the average of three independent experiments with standard deviation. wt, wild-type; hfq, *hfq* mutant.

*P. mirabilis* is capable of swarming across the surface of urinary catheters [28] and the motility ability endows *P. mirabilis* with a survival advantage, enhancing colonization within the urinary tract [28], [29]. With the notion that disruption of *hfq* impaired the cell motility in pathogenic *E. coli*, *Salmonella* and *Pseudomonas* [8], [13], [30], we thus examined the effect of Hfq on swarming and swimming abilities of *P. mirabilis*. The loss of Hfq resulted in decreased swimming and swarming motility (Figure 4A, 4B). The finding that the hfq mutant produced less flagellin, the subunit of flagella, and had few flagella (Figure 4C, 4D), is consistent with the motility result. Expression of virulence factors, including protease and hemolysin, is regulated coordinately with swarming differentiation in *P. mirabilis*. Therefore, the hemolysin activity was determined and found to be also significantly lower and increased in a delayed manner relative to the wild-type (Figure 4E) during the swarming migration process after inoculation on the swarming agar plates.

### Deletion of *hfq* affected biofilm formation, adhesion to and invasion of uroepithelial cells and fimbria production

**Figure 5:**
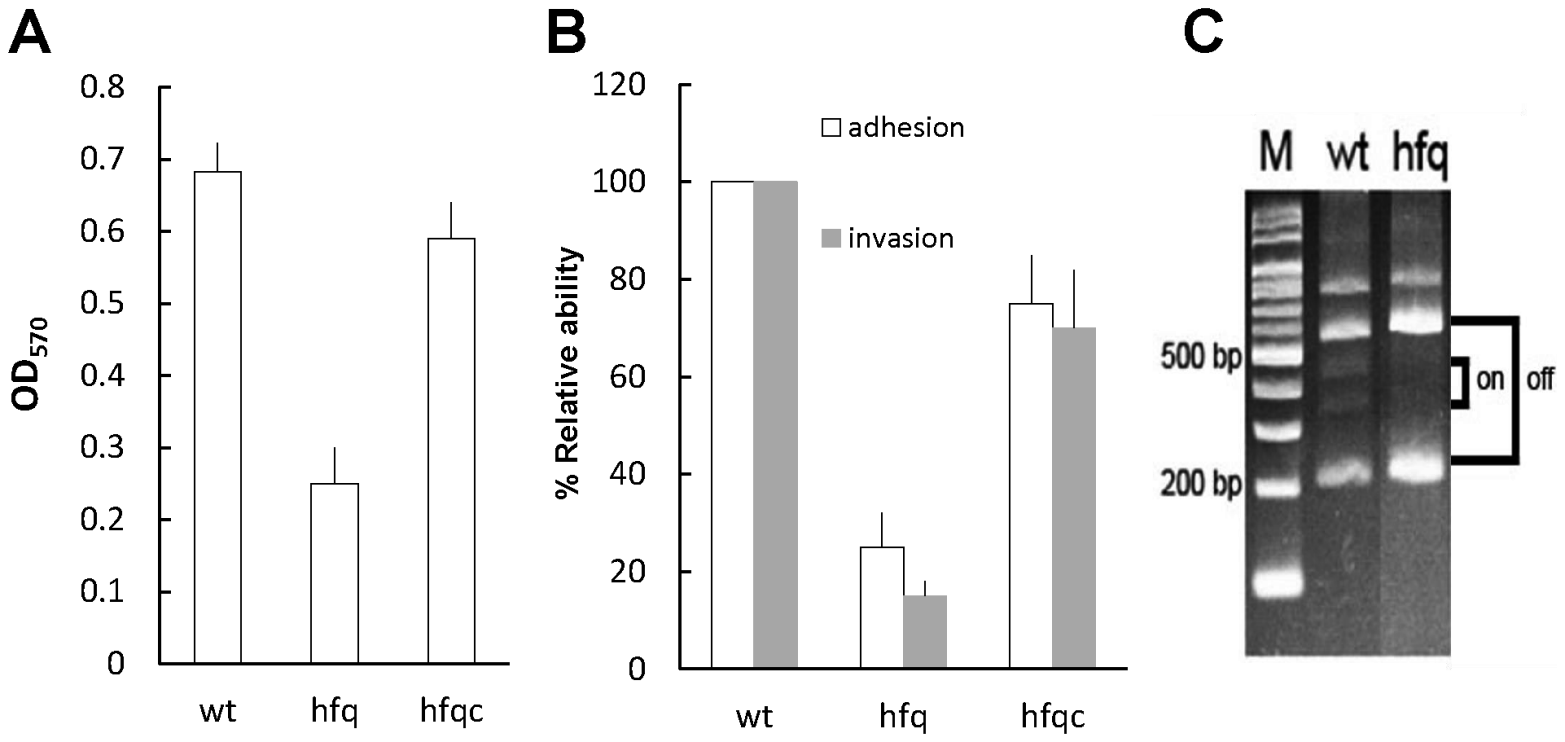
*P. mirabilis hfq* was required for biofilm formation, adhesion and invasion of uroepithelial cells and expression of MR/P fimbriae. (**A**) Biofilm formation in wild-type, *hfq* mutant and the Hfq-complemented strain. The biofilm level of the wild-type, *hfq* mutant and the Hfq-complemented strain was determined as described in Materials and Methods. The optical density (OD_570_) of the solution extracted with 95% ethanol correlated with the level of biofilm formation. (**B**) Adhesion and invasion abilities of wild-type, *hfq* mutant and the Hfq-complemented strain. Abilities to adhere to and invade NTUB1 cells were determined by assays as described in Materials and Methods. The adhesion or invasion ability of wild-type was set at 100% and other data were relative to this value. (**C**) The promoter direction of *mrp* operon in wild-type and *hfq* mutant by the invertible element assay. The assay was performed as described in Materials and Methods using overnight cultures. The “on” means direction of *mrp* promoter is for *mrp* operon expression. The data represent the average of three independent experiments with standard deviation in **A** and **B**. The representative result from three independent experiments is shown in **C**. wt, wild-type; hfq, *hfq* mutant; hfqc, Hfq-complemented strain; M, marker.

Biofilm formation can protect the pathogen from the host immune system and is essential for survival in a host [31]. *P. mirabilis* can block indwelling urethral catheters through the formation of biofilms [14]. Hence, biofilm formation was detected in this study. As depicted in Figure 5A, when *hfq* was deleted, biofilm formation was significantly decreased, in contrast to that of the wild-type. In establishing an infection, bacteria adhere to host cells, then colonize tissues, and in certain cases, invade cells for evasion of immune attacks and for subsequent bacterial persistence. Therefore, we also assessed the ability of wild-type and *hfq* mutant to adhere to and invade NTUB1 cells and found *hfq* mutant had impaired abilities to adhere to and invade NTUB1 cells (Figure 5B). As MR/P fimbriae are the surface proteins, which are expressed by *P. mirabilis* cells during infecting the urinary tract, and contribute to virulence by mediating biofilm formation and adhesion and invasion of the uroepithelial cells [32]–[34], we then examined the effect of Hfq on expression of MR/P fimbriae. Using the invertible element assay, we revealed that expression of the MR/P fimbriae was locked in the off phase in the *hfq* mutant relative to both on and off phase in the wild-type when cultured in LB broth (Figure 5C). Western blotting also showed that *hfq* mutant produced no visible MrpA protein compared to the distinct MrpA band of the wild type (data not shown). Biofilm formation, adhesion and invasion phenotypes of *hfq* mutant were restored to a certain level comparable to those of the wild type in the Hfq-complemented strain (Figure 5A, 5B).

### The effect of Hfq on expression of OMPs and RpoE in *P. mirabilis*

**Figure 6:**
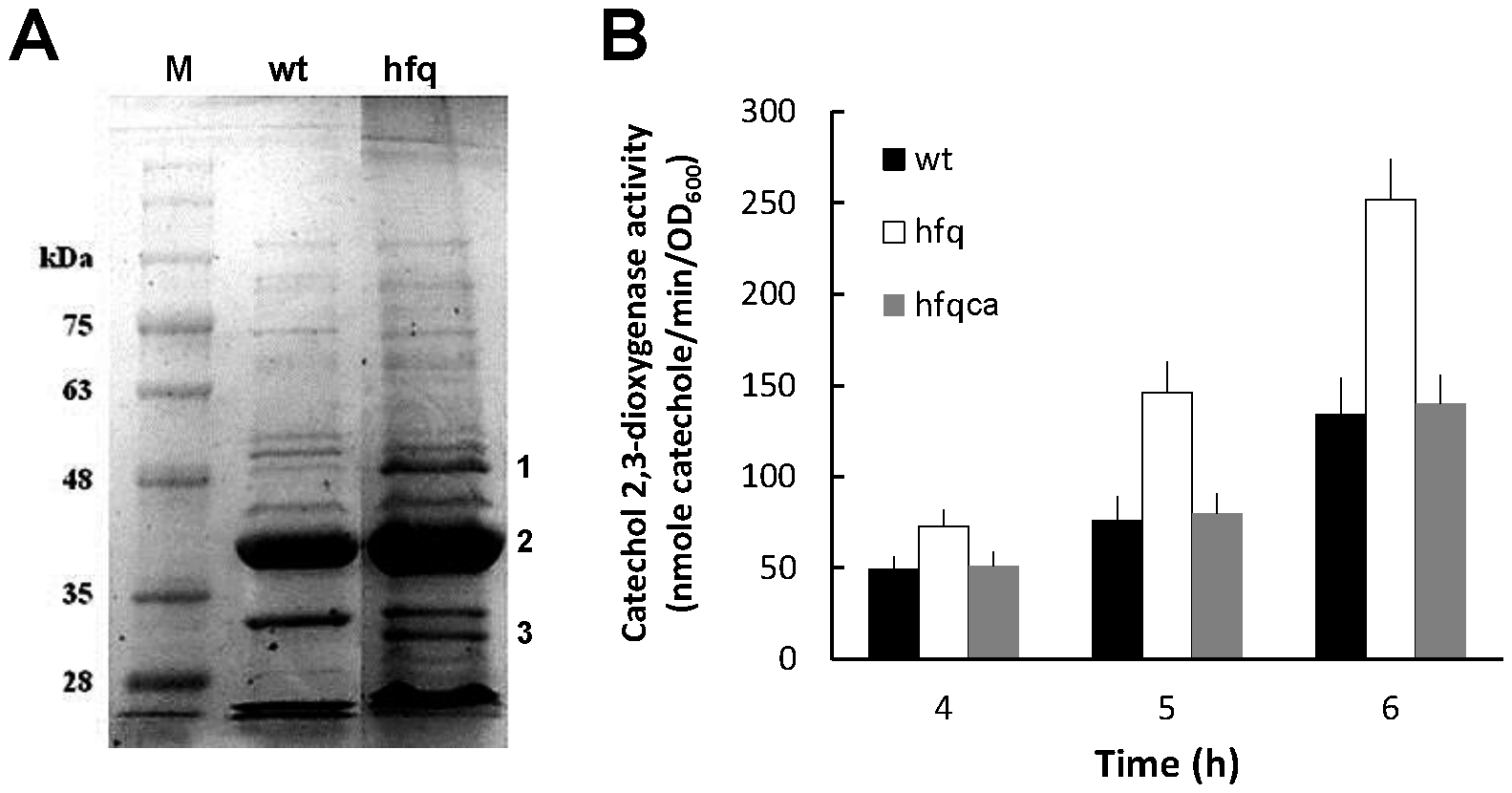
The loss of *P. mirabilis hfq* affected the OMP profile and RpoE expression. (**A**) The SDS-PAGE profile of OMPs from overnight cultures of wild-type and *hfq* mutant. The representative result from three independent experiments is shown. 1, PMI1017; 2, OmpF; 3, OmpA. (**B**) The *rpoE* promoter activities of wild-type, *hfq* mutant and Hfq-complemented strain. The activity of promoter was determined by the reporter assay in the *rpoE-xylE* reporter plasmid- transformed wild-type, *hfq* mutant and Hfq-complemented strain after incubation in LB broth for 4, 5 and 6 h. The data represent the average of three independent experiments with standard deviation. wt, wild-type; hfq, *hfq* mutant; hfqca, Hfq-complemented strain; M, marker.

As Hfq regulates OMP expression [1], [4] and a loss of Hfq has been shown to induce an envelope stress [8], [30], [35], we examined the OMP profile of wild-type and *hfq* mutant. Figure 6A reveals a different pattern of OMPs between wild-type and the mutant. Among OMP bands on the gel, PMI1017 (a putative protein belonging to the OprD family) (1), OmpF (2) and OmpA (3) were identified by MALDI-TOF mass spectrometry. Furthermore, we examined expression of the envelope stress response sigma factor, RpoE. The promoter activity of RpoE was increased in *hfq* mutant relative to wild-type after incubation in LB broth for 4, 5 and 6 h and Hfq-complemented strain (hfqca) showed a similar expression pattern to the wild-type (Figure 6B). The data indicated Hfq likely modulates OMP homeostasis and affects the envelope stress response pathway in *P. mirabilis*.

### The effect of Hfq on drug susceptibilities of *P. mirabilis*

The bacterial envelope provides a physical barrier between the cell and environment and it is crucial for bacterial growth, hence it is a key target for antibiotic discovery. As described above, Hfq affected OMP expression, a kind of envelope changes which has been shown to play a role in drug susceptibilities [36], we then test drug susceptibilities in wild-type and *hfq* mutant. As shown in Table 3, except for tetracycline, *hfq* mutant exhibited an increased susceptibility to the drugs used, ranged 2 to 16-fold increase relative to the wild-type. Moreover, the sodium dodecyl sulfate (SDS) susceptibility test was performed to evaluate the envelope disturbance. The MIC of *hfq* mutant for SDS was 32-fold lower than that of the wild type (0.025 vs 0.8%). This indicates the envelope permeability change caused by loss of Hfq may contribute to the drug susceptibility.

**Table 3:** MICs of gentamicin (Gm), streptomycin (Sm), spectinomycin (Spe), ampicillin (Amp), ciprofloxacin (Cip), tetracycline (Tc), chloramphenicol (Cm) and polymyxin B (PB) for wild-type *P. mirabilis* (wt) and *hfq* mutant (hfq).

Strain	MIC (µg/ml)							
	Gm	Sm	Spe	Amp	Cip	Tc	Cm	PB
wt	4	32	128	8	0.015625	32	16	>50000
hfq	1	2	64	4	0.0078125	32	4	4096

### Hfq controlled the level of RpoS mRNA and tolerance to H_2_O_2_ in the stationary phase seemed largely mediated through the Hfq-dependent RpoS expression

**Figure 7:**
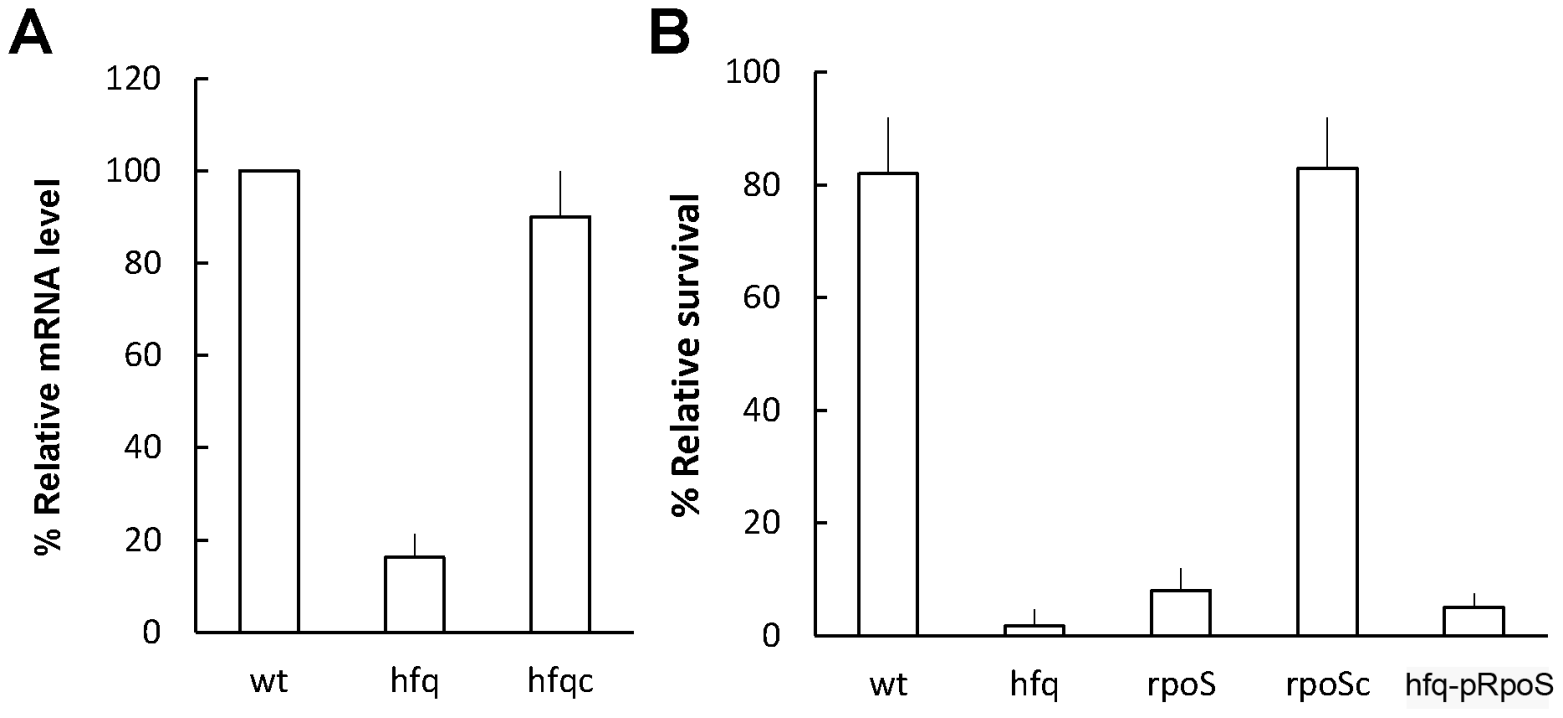
*P. mirabilis hfq* was required for the RpoS level and RpoS-expressing plasmid fails to rescue *hfq* mutant from the H_2_O_2_ killing in the stationary phase. (**A**) The level of *rpoS* mRNA in wild-type, *hfq* mutant and Hfq-complemented strain. The amounts of *rpoS* mRNA from overnight cultures of wild-type, *hfq* mutant and Hfq-complemented strain were quantified by real-time RT-PCR. The value of the wild-type was set at 100%. (**B**) The survival of wild-type, *hfq* mutant, *rpoS* mutant, RpoS-complemented strain and *hfq* mutant with the RpoS-expressing plasmid after exposure to H_2_O_2_. The H_2_O_2_ survival was determined as described in Fig. 3A except that overnight cultures was used instead of cultures of the exponential phase. All the data represent the average of three independent experiments with standard deviation. wt, wild-type; hfq, *hfq* mutant; hfqc, Hfq-complemented strain; ropS, *rpoS* mutant; rpoSc, RpoS-complemented strain; hfq-pRpoS, *hfq* mutant with the RpoS- expressing plasmid.

Hfq has been shown to regulate expression of RpoS through small non-coding RNAs [2], [37]. We found Hfq positively regulated the level of *rpoS* mRNA (Figure 7A). Consistent with the exponentially growing *hfq* mutant, *rpoS* or *hfq* mutant of the stationary phase exhibited significant reduced survival on exposure to H_2_O_2_ (Figure 3A, 7B). Moreover, an RpoS-expressing plasmid, which can compensate *rpoS* mutant for the defect in H_2_O_2_ tolerance, barely restored the H_2_O_2_ resistance of *hfq* mutant to wild-type level (Figure 7B). These data indicated the stationary phase protection of *P. mirabilis* from H_2_O_2_ is largely mediated through the RpoS pathway in an Hfq-dependent manner.

## Discussion

Recent studies revealed that Hfq, the RNA chaperone, contributes to the fitness and virulence of several pathogens [8], [10]–[13]. The spectrum and severity of *hfq* mutant phenotypes can vary among the different pathogens. For example, *Yersinia hfq* mutant is hyper-motile [38] but *hfq* mutation impairs motility in *Salmonella*, *P. aeruginosa* and *E. coli* [8], [13], [30]. Besides, deletion of *hfq* does prevent RpoS production in *Salmonella* and *E. coli* but not in *V. cholerae* [8], [11]. These results show that effects of Hfq may be unique to each bacterial species. Here, we demonstrated that Hfq plays a critical role in urinary tract colonization of *P. mirabilis* (Figure 2). *hfq* mutation led to pleiotropic phenotypic effects: changes in stress tolerance, motility, biofilm formation, invasion, the OMP profile and drug susceptibilities, were observed (Figure 3–6, Table 3).

Both swimming and swarming motility of *P. mirabilis hfq* mutant were reduced (Figure 4), consistent with the *Stenotrophomonas maltophilia hfq* mutant [19]. *Samonella* and *E. coli hfq* mutants also displayed a reduced swimming phenotype [8], [30]. In *E. coli*, the regulation of FlhDC expression by a complex network of Hfq-binding sRNAs gives cells the ability to integrate many environmental cues into the decision as to whether or not to make flagella [39]. It is reasonable to infer that there must exist Hfq-dependent sRNAs which positively regulate FlhDC expression in *P. mirabilis*, therefore production of flagellin and flagella decreases on *hfq* mutation (Figure 4). Alternatively, Hfq/sRNAs may regulate other genes of flagellum production. With this notion, *fliM*, a gene coding for the flagellar motor switch required for motility and colonization of *Helicobacter pylori*, has been shown to be regulated by a sRNA [40].

In addition to flagella, fimbriae are crucial for *P. mirabilis* to cause UTI [32], [34]. MR/P fimbriae of *P. mirabilis* mediate adhesion and invasion of the uroepithelial cells and biofilm formation [32]–[34]. For the first time, we demonstrated *P. mirabilis* Hfq is required for fimbrial production. Therefore, *hfq* mutation caused defects in cell adhesion, invasion and biofilm development (Figure 5). Implicating of Hfq in biofilm development was also observed in *E. coli* and *Salmonella* Typhimurium [30], [41].

Animal experiments showed that Hfq was required for colonization of the mouse urinary tract and infection of the rat burned skin (Figure 2A, 2B). Several lines of evidence may support the finding. First, fimbria production, biofilm formation and abilities to adhere to and invade NTUB1 cells, which are critical for UTI were impaired in *hfq* mutant (Figure 5). Second, *hfq* mutant exhibited a survival defect in macrophages (Figure 2D), which correlates to the reduced ability of *hfq* mutant to withstand the oxidative burst, simulated by the H_2_O_2_ survival test (Figure 3A). Indeed, *Y. pestis hfq* mutant, which is more sensitive to H_2_O_2_, displayed decreased intra-macrophage survival [12]. This indicates Hfq may protect *P. mirabilis* from rapid elimination by macrophages and thus for *P. mirabilis* to take advantages during the infection. With this notion, previous studies indicated that the innate susceptibility of mice to a variety of bacterial infections correlates with the ability of host macrophages to kill the intracellular (*Salmonella*) or extracellular (*E. coli*) bacteria [42]. Third, relative to wild-type, *hfq* mutant can induce higher level of IL-8 and MIF (Figure 2C), which are important cytokines to eliminate bacterial pathogens by triggering inflammatory responses [25], [26]. Knowing bacterial OMPs can stimulate IL-8 production [43], [44], it is likely that changes of OMPs (Figure 6A) caused by *hfq* mutation may induce cytokine production. *P. mirabilis* Hfq may participate in regulating expression of surface proteins that alter the immunogenicity of the bacterium or virulence factors (biofilm or swarmer cell formation) that prevent clearance by the host. Fouth, *P. mirabilis* Hfq plays a role in resistance to high osmolarity (Figure 3) and antimicrobial agents including antibacterial cationic peptide polymyxin B (Table 3). The resistance to urea is clinically relevant because urea is a major component in urine. It is likely, diverse stress tolerance of *P. mirabilis* contributes to the survival in the urinary tract, an environment with high osmolarity, and antimicrobial substances.

Previous studies have shown loss of *hfq* activates expression of the RpoE regulon [11], [30], [35] by release of RpoE from the anti-sigma factor RseA so as to trigger envelope stress responses in Gram-negative bacteria [45]. *P. mirabilis hfq* mutant appeared to experience an envelope stress condition resulting from the aberrant expression of OMPs (Figure 6A) and thereby triggered expression of the RpoE (Figure 6B). Enhanced RpoE activation probably helps to ameliorate some of the deleterious effects of *hfq* disruption. RpoE-regulated sRNAs are involved in OMP expression [46], [47] and there is ample evidence of fine tuning of bacterial OMP expression by Hfq-dependent sRNAs [35], [47]–[49]. For example, RpoE-regulated sRNAs, MicA and RybB, of *Salmonella* respond to membrane stress by accelerating OMP mRNA decay in an Hfq-dependent manner [47]. Besides, a conserved sRNA, MicM, promotes silencing of the OMP YbfM [49]. Two lines of evidence suggest that RpoE-regulated sRNAs are involved in Hfq-mediated OMP homeostasis of *P. mirabilis*. First, *P. mirabilis hfq* mutation leads to changes of OMPs and upregulation of RpoE (Figure 6A, 6B). Second, we found *P. mirabilis* RpoE positively regulated expression of RybB by the reporter assay and *hfq* mutation decreased RybB level (our unpublished data). It is worth noting that YbfM and MicM counterparts exist in the *P. mirabilis* HI4320 genome. Further analyses are underway not only to characterize the OMP targets of Hfq (PMI1017, OmpA) but also to investigate the role of RybB and MicM in *P. mirabilis*.

Relative to *rseA* mutant and wild-type, which exhibited a similar ability to colonize the mouse urinary tract, the *rpoE* mutant had significantly lower colonization of the urinary tract (our unpublished data). We found *P. mirabilis* hfq mutant had higher RpoE expression (Figure 6B) but exhibited impaired colonization of the urinary tract (Figure 2). These data indicate that both RpoE and Hfq are required for colonization. Indeed, increased RpoE in *V. cholerae hfq* mutant also doesn't account for this strain's colonization deficiency [11]. This is not surprising for RpoE-regulated sRNAs and Hfq are functional partners. It is possible that the RpoE-regulated sRNAs, in conjunction with Hfq, are critical regulators of OMP biogenesis, motility, and adherence to and invasion of uroepithelial cells, which all are important factors for *P. mirabilis* to colonize the urinary tract and cause UTI. Therefore, reduced colonization was observed in either *rpoE* or *hfq* mutant. Of course, loss of *rpoE* may be defective for entirely different reasons, leading to lower colonization.

The drug sensitivities of *P. mirabilis hfq* mutant in this study may be due to increased cell permeability or misregulation of outer membrane channels for drugs, which was supported by the increased SDS susceptibility and the OMP changes (Figure 6A), respectively. Recently, a sRNA has been shown to participate in regulating drug efflux proteins [50] and Hfq has been shown to affect drug susceptibilities by regulating expression of AcrAB efflux system [51]. Alternatively, the increased drug susceptibilities of *P. mirabilis hfq* mutant leave open the possibility that Hfq may also influence multidrug resistance by regulating efflux systems. *acrAB* genes exist in the *P. mirabilis* HI4320 genome. Investigation of the correlation of AcrAB efflux pump with Hfq/sRNAs is underway to elucidate the role of Hfq in *P. mirabilis* drug susceptibilities.

Two Hfq-dependent sRNAs, DsrA and RprA, have been demonstrated to activate the expression of *rpoS* through a mechanism whereby basepairing of the sRNAs disrupts an inhibitory secondary structure formed by the *rpoS* mRNA leader sequence and the absence of Hfq caused the reduced translation efficiency of *rpoS* [37]. The decreased transcript level of *rpoS* (Figure 7A) in *P. mirabilis hfq* mutant suggests the existence of Hfq-dependent transcriptional activators of *rpoS* or the possibility that a loss of *hfq* affects the stability of *rpoS* transcript. In this regard, *hfq* mutation in *K. pneumonia* also caused a dramatic decrease in *rpoS* mRNA [48]. Knowing production of catalase was affected by both Hfq and RpoS [13], we found either *rpoS* or *hfq* mutation resulted in a severe defect in tolerance to H_2_O_2_ (Figure 7B) accordingly. With respect to the poor restoration of H_2_O_2_ tolerance by introduction of the RpoS-expressing plasmid into the *hfq* mutant, it appears that Hfq/sRNAs are required for the translational activation of RpoS and the presence of RpoS-expressing plasmid alone fails to rescue *hfq* mutant from H_2_O_2_ killing. It's worth noting that the RprA counterpart is present in the *P. mirabilis* HI4320 genome.

To elucidate Hfq-regulatory circuits that are involved in the pathogenesis of *P. mirabilis*, co-immunoprecipitation assays should be carried out with Flag-tagged Hfq and combined with RNAseq [52] to identify all of the sRNAs that directly interact with Hfq, as well as mRNA binding partners.

In summary, this study implies Hfq as a pivotal coordinator for a diversity of regulatory circuits including surface and/or cellular components. We have demonstrated that the loss of Hfq significantly attenuates *P. mirabilis* colonization in mouse model of infection and leads to pleiotropic defects in stress tolerance, motility, invasion, biofilm formation, and resistance to antimicrobial agents. In this regard, it is tempting to suggest that Hfq may serve as a scaffold molecule for the design of novel antibacterial drugs and the *hfq* mutant is a vaccine candidate for preventing *P. mirabilis* UTI.
